# T48. ANTIPSYCHOTIC EFFICACY OF EVENAMIDE (NW-3509) IS DUE TO MODULATION OF GLUTAMATERGIC DYSREGULATION

**DOI:** 10.1093/schbul/sby016.324

**Published:** 2018-04-01

**Authors:** Ravi Anand, Emma C Forrest, Richard D Hartman, Stephen M Graham, Laura Faravelli

**Affiliations:** 1 Anand Pharma Consulting; 2 Newron Pharmaceuticals; 3 NeurWrite LLC

## Abstract

**Background:**

Over 70% of schizophrenic patients discontinue treatment with first (F)- or second-generation antipsychotics (SGA) due to dissatisfaction with their therapeutic effects; median time to discontinuation ranges from 3–7 months (1). Switching to another antipsychotic, except clozapine, did not yield better results (2). These results indicate it is essential to modulate mechanisms other than dopaminergic (DA)/serotoninergic (5-HT) systems to improve symptoms of schizophrenia (SCZ). Increasingly, NMDA receptor (NMDAr) hypofunction (3) and hippocampal hyperactivity (4) are implicated in the dysregulation of mesolimbic DA and glutamate (Glu) neurons, leading to increasing synaptic activity of Glu in the PFC (5). Augmenting the effects of current antipsychotics with Glu release inhibitors may improve symptoms of psychosis in patients with SCZ.

Evenamide does not interact with monoaminergic (DA, 5-HT, NA, H) pathways affected by current antipsychotics, or with >130 different targets involved in CNS activity, except for sodium channels, leading to modulation of Glu release. Evenamide shows efficacy in animal models of SCZ as monotherapy and as an add-on to FGA or SGA, irrespective of whether impairment was spontaneous, or induced by amphetamine, NMDAr antagonists or stress.

**Methods:**

In a pilot, proof of mechanism, randomized, double-blind, placebo-controlled, parallel group, 4-week trial, evenamide (n=50; 15–25 mg bid) or placebo (n=39) was added to patients with SCZ worsening on their current antipsychotic doses of risperidone (RIS; ≥2 mg/day) or aripiprazole (ARI; ≥10 mg/day), in 2 sites in the US (n=61) and 3 in India (n=28).

**Results:**

89 patients with SCZ (mean baseline PANSS total: 62.9 ± 7.4; CGI-S: 3.5 ± 0.5), experiencing break-through psychotic symptoms on previously effective and stable doses of RIS (mean dose: 4.2 ± 2.0 mg/day; n=70) or ARI (mean dose: 19.7 ± 7.0 mg/day; n=19) were randomized (1.3:1 ratio) to treatment with evenamide or placebo. Analyses demonstrated the addition of evenamide to RIS or ARI was associated with statistically significant efficacy, based on the PANSS Positive Symptoms sub-scale (mean change, responders), and CGI-C responder rates. The study treatments were very well tolerated; 2 patients on evenamide discontinued treatment due to AEs (atrial fibrillation and seizure). The most common AEs (evenamide vs placebo [%]), were somnolence (16 vs 12.8%), insomnia (10 vs 6%) and headache (6 vs 0%).

**Discussion:**

Addition of evenamide in patients worsening on SGAs modulating DA/5-HT significantly improved positive symptoms and CGI. No AEs such as EPS, endocrine, or sexual side effects, or weight gain were noted. These data indicate that evenamide’s Glu antagonism, demonstrated in preclinical experiments, is of value in patients worsening on current antipsychotics. Evenamide, as monotherapy or add-on, has reversed ketamine- and PCP-induced worsening of PPI. The results in the pilot clinical trial demonstrated an absence of side effects common with DA/5-HT blockers, and a rapid onset of action mediated by evenamide targeting altered Glu transmission in patients in whom SGA treatment had lost its efficacy.

Efficacy of evenamide as add-on to antipsychotics would revolutionize development of novel antipsychotics targeting aberrant firing and Glu transmission in SCZ. Potentially pivotal studies with evenamide are in planning to demonstrate that the addition of evenamide, a Glu release inhibitor, augments antipsychotic efficacy in patients worsening on current antipsychotics, and in patients with treatment-resistant SCZ not responding/worsening on clozapine.

